# Moisture-Resistant Co-Spray-Dried Netilmicin with l-Leucine as Dry Powder Inhalation for the Treatment of Respiratory Infections

**DOI:** 10.3390/pharmaceutics10040252

**Published:** 2018-12-01

**Authors:** Yingtong Cui, Xuejuan Zhang, Wen Wang, Zhengwei Huang, Ziyu Zhao, Guanlin Wang, Shihao Cai, Hui Jing, Ying Huang, Xin Pan, Chuanbin Wu

**Affiliations:** 1 School of Pharmaceutical Sciences, Sun Yat-Sen University, Guangzhou 510006, China; cuiyt3@mail2.sysu.edu.cn (Y.C.); zhanghongdou0223@126.com (X.Z.); Wen.wang@chindexmedical.com (W.W.); hzhengw3@mail2.sysu.edu.cn (Z.H.); zhaozy7@mail2.sysu.edu.cn (Z.Z.); wangglin3@mail2.sysu.edu.cn (G.W.); m13424469491@163.com (H.J.); panxin2@mail.sysu.edu.cn (X.P.); wuchuanb@mail.sysu.edu.cn (C.W.); 2 Institute for Biomedical and Pharmaceutical Sciences, Guangdong University of Technology, Guangzhou 510006, China; 3 Zhongshan School of Medicine, Sun Yat-Sen University, Guangzhou 510080, China; 4 School of Pharmaceutical Sciences, Wuhan University, Wuhan 430072, China; Alan1031csh@163.com

**Keywords:** dry powder inhalation, l-leucine, netilmicin, moisture resistant, lower respiratory tract infection

## Abstract

Netilmicin (NTM) is one of the first-line drugs for lower respiratory tract infections (LRTI) therapy, but its nephrotoxicity and ototoxicity caused by intravenous injection restrict its clinical application. Dry powder inhalation (DPI) is a popular local drug delivery system that is introduced as a solution. Due to the nature of NTM hygroscopicity that hinders its direct use through DPI, in this study, L-leucine (LL) was added into NTM dry powder to reduce its moisture absorption rate and improve its aerosolization performance. NTM DPIs were prepared using spray-drying with different LL proportions. The particle size, density, morphology, crystallinity, water content, hygroscopicity, antibacterial activity, in vitro aerosolization performance, and stability of each formulation were characterized. NTM DPIs were suitable for inhalation and amorphous with a corrugated surface. The analysis indicated that the water content and hygroscopicity were decreased with the addition of LL, whilst the antibacterial activity of NTM was maintained. The optimal formulation ND_2_ (NTM:LL = 30:1) showed high fine particle fraction values (85.14 ± 8.97%), which was 2.78-fold those of ND_0_ (100% NTM). It was stable after storage at 40 ± 2 °C, 75 ± 5% relative humidity (RH). The additional LL in NTM DPI successfully reduced the hygroscopicity and improved the aerosolization performance. NTM DPIs were proved to be a feasible and desirable approach for the treatment of LRTI.

## 1. Introduction

Lower respiratory tract infections (LRTI), mainly caused by Gram-negative (G−) bacteria of *Pseudomonas aeruginosa* (*P. aeruginosa*) or Gram-positive (G+) bacteria of *Staphylococcus aureus* (*S. aureus*), are frequently encountered in patients with cystic fibrosis or nosocomial pneumonias [1,2]. Aminoglycoside antibiotic is considered an essential LRTI therapy due to its excellent solubility, wide antimicrobial spectrum, and strong antimicrobial ability. Furthermore, it is also the first-line drug for G− bacteria and tuberculosis infections therapy. Among most aminoglycosides, netilmicin (3-*N*-ethyl sisomicin, NTM) is the first choice [3] since it exhibits the lowest nephrotoxicity and ototoxicity in clinic. NTM has been widely used in serious G− or G+ bacterial infections therapy. Also, it presents the capacity to eliminate aminoglycoside-resistant strains, particularly the bacteria that are known to possess adenylating enzymes [4]. Injection is previously the only form to administrate NTM in vivo due to its excellent water solubility and the oral non-absorption nature that related to the first-pass effect. However, NTM is unstable and easily degrades in liquid form. Besides, a large dose of NTM is required during LRTI therapy, given the complex route that NTM must take to reach the targeted site when it is administrated by injection. In this case, the large dosage may lead to dose-dependent nephrotoxicity and ototoxicity [5], restricting its further application in LRTI. Therefore, it is urgent to discover an effective NTM dosage form for LRTI therapy.

A pulmonary drug delivery system (PDDS) is a kind of local delivery system with the ability to directly deliver an active pharmaceutical ingredient (API) to the lower respiratory tract with minimum systemic toxicity [6,7]. This inspired us to enlist PDDS to deliver NTM to a targeted site, reaching the minimum inhibitory concentration (MIC) with limited plasmatic concentration. Moreover, PDDS offers a rapid onset of drug effects and satisfactory patient compliance. Nebulizers, pressured metered dose inhalers (pMDIs), and dry powder inhalation (DPI) are three common types of PDDS categories [8]. Among these, nebulizers and pMDIs are liquid forms that would make NTM unstable. Also, they can only deliver a limited dosage due to the finite drug solubility in solution and propellants. DPI as a solid system certainly exhibited the natural advantages of stability and the ability to deliver a high dose [9,10]. As mentioned, a substantial amount NTM is needed for LRTI therapy to grant a promising DPI approach.

**Figure 1:**
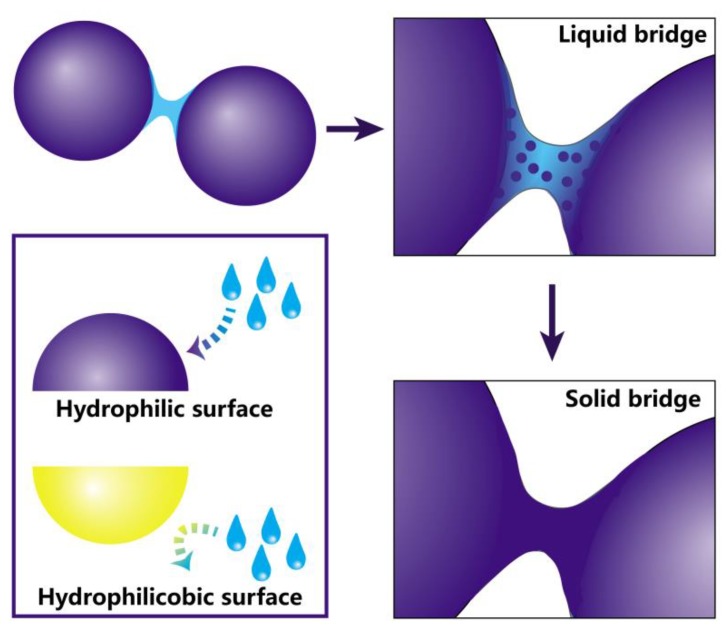
The moisture absorption process of particles.

However, the nature of NTM hygroscopicity, its application through DPI is restricted. The increased particle size, causing by hygroscopicity in DPI formulations during storage, ultimately deteriorates the aerosolization performance [11,12]. The moisture absorption process is shown in Figure 1. NTM with hydrophilic surface was most likely absorbing moisture; the liquid bridges formed between NTM particles finally develop into solid bridges that induce particle agglomeration, evidently changing the physicochemical properties and increasing the inter-particulate force of NTM [13]. When this force between particles becomes higher, particles easily cohere and agglomerate, leading to a poor aerosolization performance. Moreover, the absorbed moisture accelerates the NTM hydrolytic degradation speed, causing a harmful impact on the efficacy of NTM [14].

Adding carriers to DPI would effectively reduce its hygroscopicity. Normally, there are two types of carrier-based DPI. First, the carrier and drug are physically mixed to improve the flowability and dispersibility [15,16,17]. Second, the carrier and drug are dissolved and co-spray-dried [18,19,20]. However, the added carrier amount is generally large. As we know, the dosage of DPI delivered to the lung is relatively finite compared to oral administration or intravenous injection, while the dosage of NTM is large. The NTM proportion would decrease in DPI as the carrier proportion increases. Therefore, the incremental dosage caused by the added carriers becomes undesirable for NTM DPIs.

l-leucine (LL) was introduced into NTM DPIs to overcome their hygroscopicity in this study. LL is a surfactant with both hydrophobic and hydrophilic groups, and is widely used as a safe excipient in pharmaceutics [21,22,23]. It is assumed that LL works as a surfactant and enriches the particles surface during the spray-drying process, generating a hydrophobic surface [24]. As mentioned above, solid surface hydrophilicity plays a key role in moisture uptake process. The hydrophobic surface formed by LL could reduce the hydroscopic growth of NTM particles [25]. It was reported that an additional 10% of LL effectively enhances the aerosolization performance of highly hydroscopic DPIs [26]. For NTM DPIs, the lowest added LL amount that achieves moisture resistance should be considered, owing to the relatively high dosage of NTM.

Moreover, LL may change the morphology of NTM particles [24]. The morphology of particles has an important effect on DPI aerosolization performance; for example, particles with appropriate corrugation could have enhanced flowability and improved aerosolization performance [27]. As a consequence, the influence of the addition amount to the particle morphology should be considered.

The purpose of this study was to develop an NTM DPI with moisture resistance, enhanced flowability, and improved aerosolization performance. NTM was co-spray-dried with different amounts of LL, and the particle size, morphology, crystallinity, water content, hygroscopicity, in vitro aerosolization performance, as well as stability of the DPIs were evaluated.

## 2. Materials and Methods

### 2.1. Materials

Netilmicin Sulfate was obtained from Beierka Biopharmaceutical Co., Ltd. (Wuhan, China). l-leucine was purchased from Macklin Inc. (Shanghai, China). Trifluoroacetic acid was obtained from Shanghai Aladdin Reagent Co., Ltd. (Shanghai, China). Methanol was supplied by Honeywell Burdick & Jackson Inc. (Morris, NJ, USA). *Pseudomonas aeruginosa* (ATCC9027), *Escherichia coli* (*E. coli*, ATCC9739), and *Staphylococcus aureus* (ATCC6538) were purchased from Guangdong Microbiological Detection Center (Guangdong, China). Mueller–Hinton broth medium was supplied by Thermo Fisher Oxoid (Basingstoke, UK). All other reagents were used as received.

### 2.2. Preparation of NTM DPIs by Spray-Drying

The spray-drying (SD1000 spray-drying, Eyela Co., Ltd., Tokyo, Japan) was used to fabricated NTM DPIs under the following conditions: inlet temperature 130 °C, outlet temperature 75 °C, air flow 0.7 m^3^/min, atomization pressure 190 kPa, feed flow rate 1 mL/min. The above conditions were selected by preliminary experiments. Feed solutions were prepared by dissolving NTM and LL in ultrapure water with different mass ratios (Table 1) with a feed concentration (total solids content) of 30 mg/mL. In addition, spray-dried NTM without LL served as a reference (ND_0_).

### 2.3. Particle Size of NTM DPIs

The particle size of the NTM DPIs was analyzed by a laser diffraction particle size analyzer (Malvern Mastersizer 2000, Malvern Instruments Ltd., Malvern, Worcestershire, UK) with the associated software. The particle was dispersed by a Scirocco 2000 dry powder feeder under an air pressure of 3.5 bar airstream. Each formulation was measured in triplicate.

### 2.4. Bulk Density and Tap Density

A reported method [28] was conducted to test the bulk density (*ρ*_b_) and tap density (*ρ*_t_) of each formulation. Precisely weighed powder was loaded into a cylinder. The mass and volume of the powder were recorded as *m* and *V*_b_, respectively. Subsequently, the cylinder was tapped at least 300 times, until no obvious change in volume was observed, and the final volume (*V*_t_) was recorded. *ρ*_b_ and *ρ*_t_ are calculated using Equations (1) and (2):

(1) $$\rho_{b}=\frac{m}{V_{0}}$$

(2) $$\rho_{t}=\frac{m}{V_{t}}$$

### 2.5. Scanning Electron Microscopy (SEM)

SEM (Gemini 500 scanning electron microscope, Bruker, Germany) was adopted to characterize the surface morphology of particles. Sample powders were placed on aluminum stubs prior to imaging. The images were captured at an acceleration voltage of 1.0 kV.

### 2.6. Powder X-ray Diffraction (PXRD)

Crystallinity was evaluated by PXRD (D-MAX 2000 VPC, Rigaku, Japan). The sample was spread into the cavity of the PXRD and then analyzed by Cu Kα radiation (30 mA, 40 kV) from 3° to 40°, at a step rate of 0.2°/s.

### 2.7. Water Content Measurement

Water content analysis was conducted by a Karl–Fisher titration. The measurements were performed with a 756 KF coulometer (Metrohm Ltd., Antwerp, Belgium). The reaction (Equation (3)) involved in the Karl–Fisher titration was as follows:

(3) $$I_{2}+SO_{2}+2H_{2}O=2HI+H_{2}SO_{4}$$

### 2.8. Hygroscopicity Investigation

Powders were precisely weighed for each formulation (*m*_1_), placed in inert glass containers, and stored at the simulated climate cabinet (CLIMACELL 222t, 3M Medcenter Einrichtungen GmbH, Planegg, Germany) under 25 ± 1 °C and 80% RH. The NTM DPIs were directly exposed to the humidity. After 24 h of storage, the containers were taken out and weighed again (*m*_2_). The weight gain % of each formulation was calculated using Equation (4):

(4) $$\mathrm{Weight}\;\mathrm{gain}\;\%=\frac{m_{2}-m_{1}}{m_{1}}\times 100\%,$$

### 2.9. Antibacterial Assay of NTM DPIs

The MIC was defined as the lowest antibiotic concentration that inhibited a visible planktonic bacterial growth. Optical density (OD) was measured at 600 nm by a microplate reader (EL×800, Biotek, Winooski, VT, USA), which was used to examine the visible bacterial growth. Briefly, a single colony of *P. aeruginosa*, *E. coli*, and *S. aureus* were transferred to 5 mL Mueller–Hinton broth medium with 150 rpm shaking at 37 °C for 12 h. The concentrations of bacteria were measured by a bacterial turbid meter (WGX-2-XJ, Xinrui Ltd., Shanghai, China). The relationship between the colony forming unit (CFU) of the bacteria and the maid turbidity unit (MCF) is expressed as:

(5) $$1\;\mathrm{MCF}=3\times 10^{8}\;\mathrm{CFU},$$

The bacteria suspension with 0.15 MCF was further diluted by 100-fold. Different formulations were diluted in phosphate buffer saline to prepare 400 μg/mL NTM DPIs solutions. Then, 50 μL NTM DPIs solutions were added to 50 μL diluted bacteria suspensions (4.5 $\times 10^{5}$ CFU) to achieve a final NTM DPI concentration of 200.0, 100.0, 50.00, 25.00, 12.50, 6.250, 3.125, 1.563, 0.7813 μg/mL. The samples were incubated at 37 °C for 12 h. The lowest concentration that yielded OD_600_ ≤ 0.1 was determined as the MIC.

### 2.10. Qualification by HPLC

The samples obtained from in vitro aerosolization performance examination were quantified by high performance liquid chromatography (HPLC-2010, Shimadzu, Japan) with an evaporative light-scattering detector (ELSD-LT II, Shimadzu, Japan), using a C18 column (Phenomenex, Gemini, 4.6 mm × 250 mm, 5 μm). The mobile phase was 0.2 mol/L trifluoroacetic acid–methanol (84:16, *v*/*v*), and the flow rate was at 0.5 mL/min. The temperature of the drift tube and gas pressure of the detector were set at 40 °C and 350 kPa, respectively. The calibration curves were linear (*R*^2^ = 0.9995) over the concentration range of 1 to 40 μg/mL. Each sample was quantified in triplicate.

### 2.11. In Vitro Aerosolization Performance Examination

The in vitro aerosolization performance of NTM DPI was evaluated by a twin stage impinger [29] (TSI, National Center for Pharmaceutical Engineering, Shanghai, China) and a Turbospin^®^ device (PH&T S.p.A., Milan, Italy) was selected as the inhaler. Firstly, the HPLC mobile phase was added to the upper stage (7 mL) and lower stage (30 mL) of the TSI. NTM DPIs were loaded into 3# Vcaps^®^ capsules (Capsugel Co., Ltd., Suzhou, China) with 30.0 ± 0.5 mg. Then, the capsule was inserted into the pulverization chamber of the Turbospin^®^ vertically and a needle pierced the bottom of the capsule. The inhaled air was introduced and emptied the capsule. The vacuum pump was operated at a flow rate of 60 L/min for 5 s for each capsule. Each in vitro aerosolization experiment was performed on three capsules. Then, the TSI was dismantled and the amount of NTM deposited in Stage 1 and Stage 2 of the TSI was measured by the HPLC method described above. Also, the inhaler and the TSI throat were rinsed by the HPLC mobile phase and analyzed by the same method. The recovered dose (RD) represented the total mass of the NTM detected (Equation (6)). The fine particle dose (FPD) was defined as the amount of NTM recovered from Stage 2 with a cut-off diameter of 6.4 μm. The fine particle fraction (FPF) represented the percentage of particles with an aerodynamic diameter of less than 6.4 μm used to evaluate the aerosolization performance of NTM DPIs [30]. The FPF values were calculated using Equation (7):

(6) RD=Inhaler+Throat+Stage 1+Stage 2,

(7) $${\mathrm{FPF}}_{<\;6.4\;\mu m}=\frac{\mathrm{FPD}}{\mathrm{RD}}\times 100\%,$$

### 2.12. Stability Under Accelerated Conditions

The optimal formulation was loaded into 3# Vcaps^®^ capsules with 30.0 ± 0.5 mg and placed in a 15-mL glass penicillin bottle without a lid, then stored in the stability chamber (Climatic and Thermostatic Chamber Mod. CCP37, AMT srl, MI, Italy) at 40 ± 2 °C, 75 ± 5% RH for 3 months. The appearance of the DPI in capsules was observed. The stability was assessed by the TSI test as described above.

### 2.13. Statistical Analysis

An ANOVA test was then conducted on the experimental data obtained above. Significant differences between formulations were analyzed using post-hoc multiple comparisons, where *p* < 0.05 was considered to be statistically significant.

## 3. Results and Discussion

### 3.1. Particle Size of NTM DPIs

Particle size distribution parameters of NTM DPIs were listed in Table 2. The *d*_0.5_ values of each formulation were smaller than 5 μm that was potential to be used in DPIs. A significant difference (*p* < 0.05) in *d*_0.5_ could be discovered, and ND_2_ possessed the smallest *d*_0.5_ values among NTM DPIs. The *D*_4.3_ values of NTM DPIs were larger than 3 μm, enabling them to easily escape the capture of macrophages and show antibacterial activity in the deep pulmonary in vivo.

### 3.2. Bulk Density and Tap Density

**Figure 2:**
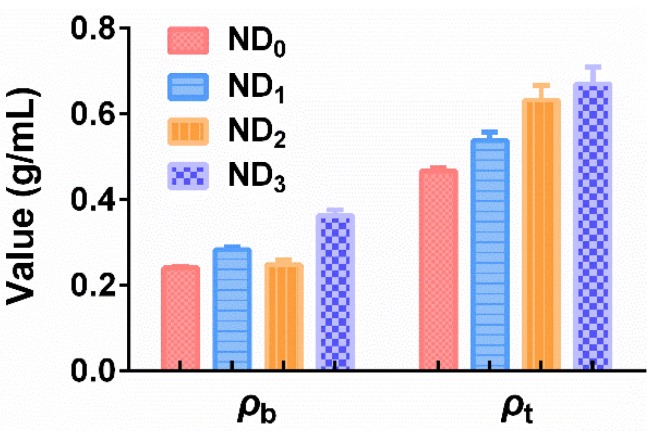
The bulk density and tap density of NTM DPIs (*n* = 3).

The *ρ*_b_ and *ρ*_t_ of ND_0_~ND_3_ are shown in Figure 2. For *ρ*_b_, no significant difference (*p* > 0.05) was observed between ND_0_ and ND_2_; they both possessed the lowest *ρ*_b_ among NTM DPIs. Previous studies from our group demonstrated that particles with similar diameters but lower *ρ*_b_ possessed better aerosolization performance and higher FPF values [15,31]. They were more sensitive to air flow and more easily fluidized during inhalation. Also, they encountered less gravity, which caused less deposition in the upper respiratory tract; thus, the drug was capable of reaching deeper sites in the pulmonary. As for *ρ*_t_, it became higher as the LL proportion increased.

The aerodynamic diameter (*d*_ae_), which was applied to characterize the aerodynamic properties of particles [32,33], represented the inherent tendency of aerosol deposition in both gravitational settling and inertial impaction [34]. Aerosolization performance was influenced by multiple powder properties such as particle size [33], density [15], shape [35], and surface roughness [20], etc. Besides, *d*_ae_ is affected by several factors including density and shape. Hence, *d*_ae_ could better characterize the actual process of drug deposition. *d*_ae_ could be related to the particle diameter and density, as expressed in Equation (8).

(8) $$d_{\mathrm{ae}}=\sqrt{\frac{\rho }{\rho_{0}\;x}}d$$

where *ρ* represents the bulk density, *ρ*_0_ denotes the unit density (1 g/cm^3^), *χ* represents the shape factor, and *d* is the diameter of the particle.

The results (Table 2) showed that the *d*_ae_ values of NTM DPIs were in the range of 1~3 μm, indicating they could reach the alveoli, which is desirable for pulmonary delivery [36]. Among them, ND_2_ with the smallest *d*_ae_ was more easily fluidized and dispersed in the air flow [37], getting through the bronchioles and finally being deposited on the alveoli [38,39]. Therefore, it was speculated that ND_2_ may have the best aerosolization performance. Meanwhile, ND_3_ with the largest *d*_ae_ might possess the worst aerosolization performance among all the formulations [36].

### 3.3. Scanning Electron Microscopy (SEM)

**Figure 3:**
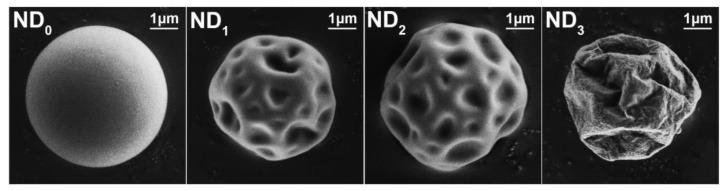
SEM images of NTM DPIs.

The morphology of spray-dried NTM is shown in Figure 3. Spray-dried NTM (ND_0_) exhibited a spherical shape with a smooth surface whereas the NTM co-spray-dried with LL (ND_1_~ND_3_) showed a spheroid but corrugated morphology. It is believed that the additional LL was responsible for the particle morphology alternation. Particle corrugation was even more obvious and the wrinkles were more distinct with increased proportions of LL.

**Figure 4:**
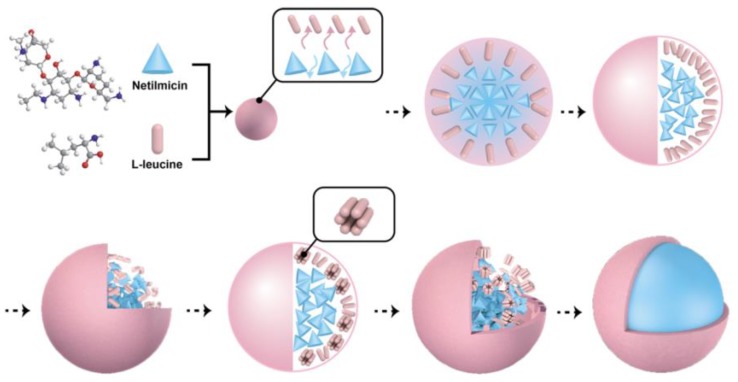
Schematic illustration of the formation mechanism of NTM DPIs.

A hypothesis of the mechanism of corrugated NTM DPIs particles formation is presented in Figure 4. As soon as the NTM and LL droplet was exposed to the spray-drying heat flow, water evaporation took place at the gas–liquid interface and gradually a solid shell as well as a gas–solid interface was formed. During the water evaporation at the gas–solid interface, there was a water concentration gradient in the droplet. As a result, water migrated from the droplet liquid core to the solid shell and the distribution of NTM and LL was driven by the water migration force, which dragged them from the inner part to the outer part of the droplet.

For binary systems, the hydrophilicity and the molecular weight of each component could influence their distribution during the drying process [1]. LL, as an amphipathic molecule and a surfactant, was able to form micelles in the solvent. Although the LL concentrations in the feeding solutions were far lower than the previously reported critical micelle concentration (CMC) range (0.015~0.023 mM) [40], the LL concentrations would still increase quickly during solvent evaporation and achieve CMC. Under this circumstance, LL micelles formed and showed surface activity that caused LL to concentrate at the droplet surface with an outward hydrophobic group and decreased surface tension. On the contrary, hydrophilic NTM was prone to remain in the inner part of the moisture droplets. Furthermore, the molecular weight of NTM (1441.54 g/mol) was about 10-fold that of LL (131.18 g/mol). Thus, LL encountered less resistance when it diffused within water and possessed a superior migration rate during the drying process. 

Through a comprehensive consideration of various factors, LL was enriched on the external surface of the droplet and formed the shell with less permeability, causing slow solvent evaporation. The solid–liquid interfacial tension could not maintain the spherical skeleton, which allowed the particle to exhibit a corrugated morphology. Meanwhile, when the solvent evaporated quickly, the hydrophilic NTM formed particles with smooth surfaces. Evidently, micrographs of samples containing different LL proportions confirmed that the effect of hydrophobic excipients concentrated on particle morphology.

### 3.4. Powder X-ray Diffraction (PXRD)

**Figure 5:**
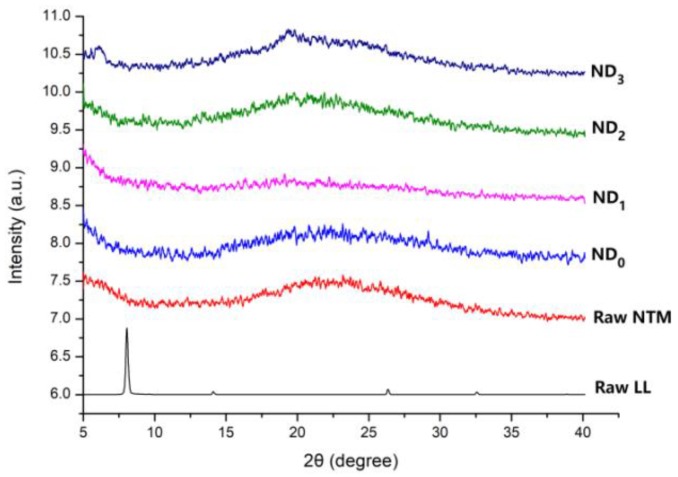
Powder X-ray diffraction (PXRD) patterns of raw NTM, raw l-leucine (LL), and NTM DPIs.

PXRD diffractograms of each NTM DPI formulation are presented in Figure 5. Raw LL exhibited clear crystalline peaks at 8°, while spray-dried NTM with LL and raw NTM showed no obvious peak, demonstrating that all the formulations prepared by spray-drying exhibited an amorphous form.

### 3.5. Water Content and Hygroscopicity Test

**Figure 6:**
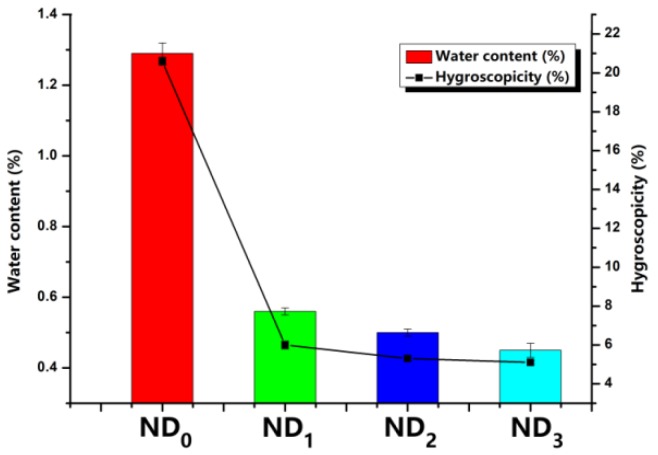
Water content and hygroscopicity results of different NTM DPIs (*n* = 3).

Water content and hygroscopicity data are shown in Figure 6. With the addition of LL, the water content of NTM DPIs decreased from 1.29% to 0.56%. It was assumed that LL could guard against moisture penetration due to its enrichment on the particle surface. Also, the effects of LL on the hygroscopicity of all formulations were clearly observed. Hygroscopicity was detected by utilizing the weight gain after storage at 80% RH for 24 h; the different NTM DPIs showed different levels of hygroscopicity. Specifically, ND_0_ presented the highest weight gain of 20.6 ± 0.2%. When co-spray-dried with LL, the weight gain of NTM DPIs dropped to 5.3 ± 0.1%. It was demonstrated that the appropriate additional amount of LL could prevent the moisture absorption of NTM.

The moisture uptake of pure NTM (ND_0_) could be caused by capillary forces and/or recrystallization, inducing particles agglomeration and poor flowability. Moisture could increase the thickness of the absorbed liquid layer and strengthen the liquid bridges between particles [41]. Therefore, it would be easier for particles to cohere and agglomerate [42]. As a result, the flowability would deteriorate with the increment of moisture uptake. Previous studies had reported that the hydrophobic surface of particles certainly affected the powders hygroscopicity, which prolonged the powders deliquesce period, greatly reducing particle agglomeration [26,43,44]. Hence, hydrophobic LL enrichments on each particle surface could inhibit agglomeration. According to the SEM, the effect of LL on hygroscopicity could be attributed to the LL distribution on the NTM particles surface, resulting in less diffusion of moisture through the LL surface. In summary, LL was demonstrated to enhance the moisture resistance of NTM DPIs.

### 3.6. Antibacterial Assay of NTM DPIs

**Figure 7:**
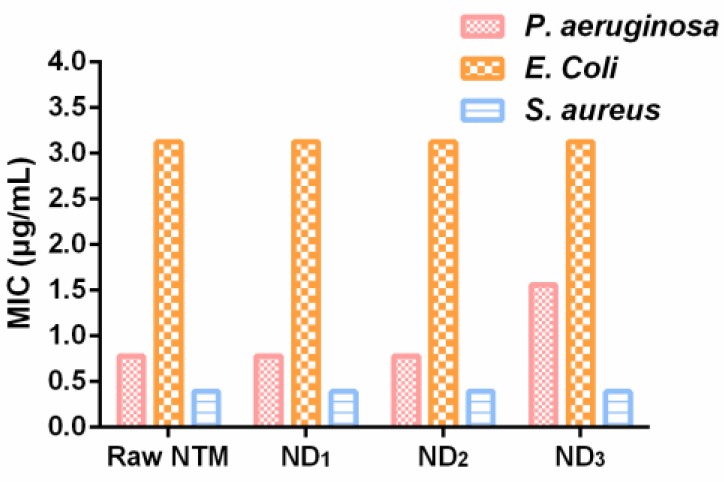
Minimum inhibitory concentrations of different NTM DPIs (*n* = 6).

The MIC values of NTM DPIs are presented in Figure 7. No evident changes in the antibacterial activity were showed with the addition of LL. To be specific, the MIC values of ND_1_ and ND_2_ were consistent with that of raw NTM. The ND_3_ contained about 10% LL and the MIC values of *P. aeruginosa* were slightly larger than those of other DPI formulations. It was assumed that *PA* was more sensitive to NTM compared with *E. coli* and *S. aureus*. When the NTM proportion was reduced, the *P. aeruginosa* bacteriostatic ability was decreased. On the contrary, *E. coli* and *S. aureus* were not that susceptible to NTM; hence, the decreased NTM amount did not affect the MIC.

### 3.7. In Vitro Aerosolization Performance Examination

The TSI experiments (Table 3) showed that the RD values of ND_1_~ND_3_ were in the range of 82.7% to 98.2%, whilst ND_0_ presented the minimum values (69.3 ± 2.2%). This demonstrated that more than 30% of ND_0_ was retained within the capsule and the inhaler device. Furthermore, the LL concentration apparently affected the variation of the FPF values. When the LL proportion increased approximately 3% (*w*/*w*) from the beginning, the FPF values became correspondingly higher, indicating an improved aerosolization performance of DPIs. The ND_2_ obtained the highest FPF values (85.14 ± 8.97%), identified using the results of the *d*_ae_ calculation. However, the tendency of the FPF values was not consistent with the *d*_ae_ result. This might be associated with the water content and hygroscopicity of NTM DPIs. As previously stated, ND_0_ possessed the highest water content and hygroscopicity, resulting in the worst flowability accompanied with the lowest RD values. The capabilities of LL that formed a hydrophobic corrugated surface and reduced aggregation improved this situation. The presented results were consistent with previous reports about surface corrugation. Particles with a hydrophobic corrugation morphology encountered less inter-particulate cohesion since the van der Waals forces were reduced and, consequently, the powder reparability and the aerosolization performance of particles were improved [45,46]. Moreover, the calculation of *d*_ae_ was idealized, neglecting the corrugated particles morphology.

**Figure 8:**
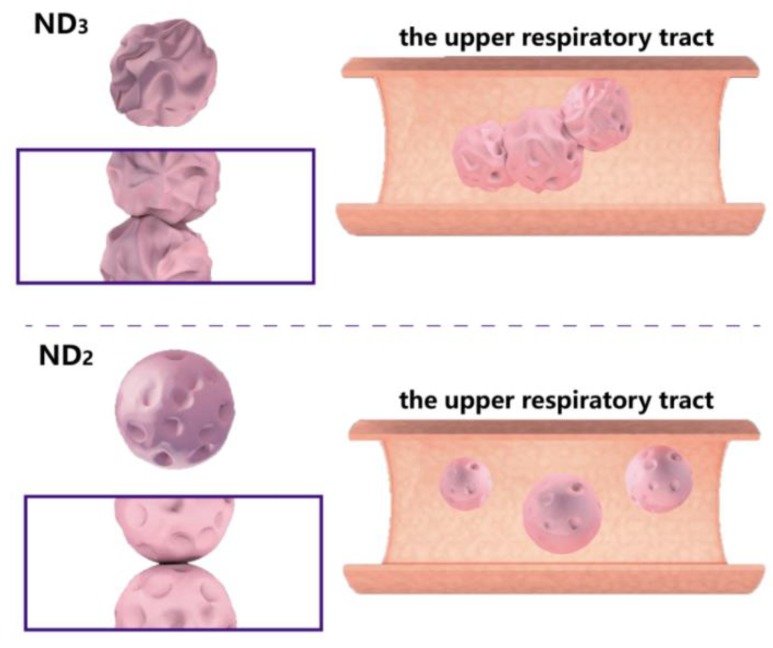
The influence of a corrugated particle surface in the respiratory tract.

It is worth noting that the RD and the FPF values declined sharply as the LL proportion increased (ND_3_). This may be related to the over-wrinkled surface of the ND_3_ particles, confirmed by the SEM. The surface morphology had an influence on *ρ*_b_ that further affected the FPF values. Corrugation was more obvious with increasing amounts of LL (Figure 3), and the corrugated surface led to a lower *ρ*_b_. However, the over-corrugated surface of ND_3_ might cause the particles to become embedded in each other (Figure 8). In this case, the space between particles decreased and *ρ*_b_ increased as a result. ND_2_, with a rational corrugated surface, had lower *ρ_b_* and higher FPF values. Moreover, particles with an over-corrugated surface were embedded in each other; the cohesion force between particles became stronger, causing agglomeration. Besides, particles with an over-corrugated surface encountered stronger air flow resistance, which led to poor flowability and thus they could not be fully fluidized [31,47]. Ultimately, they were deposited in the capsules, inhaler device, and upper respiratory tract, thus generating lower RD and FPF values. On the contrary, ND_2_ exhibited a rational extent of corrugation that was not deep enough to embed other particles. Meanwhile, the suitable corrugation morphology lessened the cohesion force, allowing particles to pass through the device successfully and granting the highest RD and FPF values. In conclusion, LL improved the aerosolization performance by forming a hydrophobic and appropriately corrugated surface. Among all the formulations, ND_2_ showed the optimal RD values (98.2 ± 0.2%) and FPF values (85.14 ± 8.97%), and thus presented the most satisfactory pulmonary deposition ability.

### 3.8. Stability under Accelerated Conditions

The optimal formulation (ND_2_) was evaluated to be stable at 40 ± 2 °C, 75 ± 5% RH. The results of TSI are shown in Table 4, suggesting that the samples had no significant difference in RD and FPF as compared with the control (*p* > 0.05). Moreover, in appearance, all samples seemed to be the same as the control. Thus, these data confirmed that the NTM co-spray-dried with LL was humidity resistant; the NTM DPIs were able to maintain their aerosolization performance after 3 months of storage at an accelerated condition.

## 4. Conclusions

LRTI are associated with a high mortality rate, and thus demand effective antibiotic treatment. However, the toxicity of NTM restricts its clinical application during long-term therapy. DPI, as a local drug delivery formulation, can deliver NTM to the infection site directly with minimum toxicity, high local concentration, and low plasmatic concentration. LL is a hydrophobic amino acid that was introduced into NTM DPIs to increase the moisture resistance and improve the aerosolization performance.

Additional LL within the NTM DPIs significantly reduced the water content and hygroscopicity. LL, as an aerosol enhancer, provided significant moisture protection to particles by forming a hydrophobic surface. The optimal formulation (ND_2_) presented 85.14% FPF value improvements while maintaining full antibacterial activity. In summary, NTM DPIs were successfully developed as a feasible and desirable system to treat LRTI in this research. It is anticipated that this novel dosage form will be widely used for LRTI symptom relief while also improving therapeutic outcomes.

## Figures and Tables

**Table 1:** Composition of netilmicin (NTM) dry powder inhalations (DPIs).

Formulation	ND_0_	ND_1_	ND_2_	ND_3_
NTM:LL	100:0	50:1	30:1	10:1

**Table 2:** Particle size of NTM DPIs (*n* = 3).

Formulation	*d*_0.1_ (μm)	*d*_0.5_ (μm)	*d*_0.9_ (μm)	*D*_4.3_ (μm)	Span	*d*_ae_ (μm)
ND_0_	1.46 ± 0.04	3.07 ± 0.12	6.60 ± 0.99	7.56 ± 3.23	2.42 ± 0.01	1.50 ± 0.07
ND_1_	1.39 ± 0.01	3.07 ± 0.04	6.05 ± 0.07	3.44 ± 0.04	2.35 ± 0.01	1.63 ± 0.04
ND_2_	1.36 ± 0.01	2.89 ± 0.02	5.42 ± 0.04	3.17 ± 0.02	2.40 ± 0.01	1.44 ± 0.05
ND_3_	1.36 ± 0.01	3.07 ± 0.01	6.00 ± 0.03	3.41 ± 0.01	2.38 ± 0.01	1.84 ± 0.03

**Table 3:** Aerosolization performance of NTM DPIs (*n* = 3).

Formulation	RD ^a^ (%)	FPF ^b^ (%)
ND_0_	69.3 ± 2.2	30.60 ± 7.56
ND_1_	93.3 ± 1.7	64.93 ± 6.36
ND_2_	98.2 ± 1.2	85.14 ± 8.97
ND_3_	82.7 ± 1.8	47.82 ± 7.44

^a^ RD, recovery dose; ^b^ FPF, fine particle fraction.

**Table 4:** Aerodynamic behavior of ND_2_ after storage in an accelerated condition (*n* = 3).

Month(s) of Storage	RD (%)	FPF (%)
0	98.2 ± 1.2	85.14 ± 8.97
1	97.9 ± 1.6	83.26 ± 5.88
2	97.7 ± 1.4	84.78 ± 6.49
3	97.3 ± 2.0	83.31 ± 6.72
